# Some reflections on the understanding of the oxygen reduction reaction at Pt(111)

**DOI:** 10.3762/bjnano.4.108

**Published:** 2013-12-27

**Authors:** Ana M Gómez-Marín, Ruben Rizo, Juan M Feliu

**Affiliations:** 1 Instituto de Electroquímica, Universidad de Alicante, Apt. 99, Alicante, E-03080, Spain

**Keywords:** hydrogen peroxide oxidation, hydrogen peroxide reduction, oxygen reduction, Pt(111), stepped surfaces

## Abstract

The oxygen reduction reaction (ORR) is a pivotal process in electrochemistry. Unfortunately, after decades of intensive research, a fundamental knowledge about its reaction mechanism is still lacking. In this paper, a global and critical view on the most important experimental and theoretical results regarding the ORR on Pt(111) and its vicinal surfaces, in both acidic and alkaline media, is taken. Phenomena such as the ORR surface structure sensitivity and the lack of a reduction current at high potentials are discussed in the light of the surface oxidation and disordering processes and the possible relevance of the hydrogen peroxide reduction and oxidation reactions in the ORR mechanism. The necessity to build precise and realistic reaction models, which are deducted from reliable experimental results that need to be carefully taken under strict working conditions is shown. Therefore, progress in the understanding of this important reaction on a molecular level, and the choice of the right approach for the design of the electrocatalysts for fuel-cell cathodes is only possible through a cooperative approach between theory and experiments.

## Introduction

**Scheme 1:**
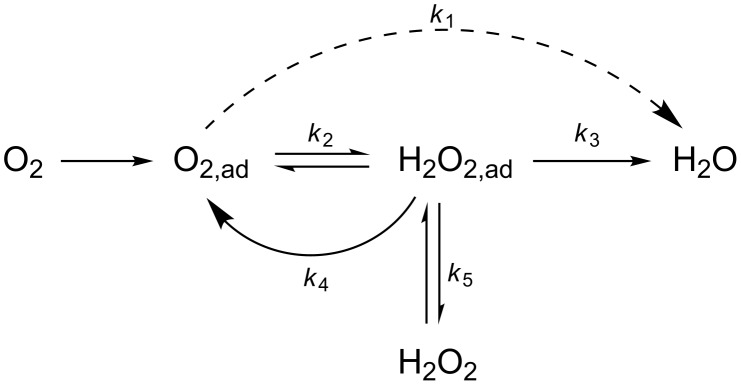
Reaction pathways proposed for the ORR given by Wroblowa et al. [1].

Nowadays, the oxygen reduction reaction (ORR) is arguably one of the most important challenges in electrocatalysis and it is undoubtedly the most important cathodic process in fuel cells. It is a complex 4-electron reaction that involves the breaking of a double bond and the formation of 4 OH-bonds through several elementary steps and intermediate species. A generally accepted, classical scheme for this reaction, in which hydrogen peroxide is a stable reaction intermediate species, can be depicted by Scheme 1 [1].

However, despite the intensive experimental and theoretical ORR research for decades, which ranges from studies on idealized model electrodes up to reaction studies in technical systems, up to now the exact ORR mechanism is not clearly known. This has different reasons. First, the currently available experimental techniques are not capable of detecting all possible reaction intermediates in a complex process such as the ORR. Second, the reaction takes place at high overpotentials and the activation region is quite limited to the onset of the wave. Thus, the transport-controlled region appears very soon, which limits the potential range, in which the electron transfer mechanism can really be studied. Therefore other approaches are needed that include theoretical calculations that could help to shed light on the microscopic structures and processes taking place at the surface during the reaction.

As starting point, quantum chemical models consider perfectly ideal materials. This means that, within the state of the art of the calculation, surfaces have no defects and atoms at the surface correspond to the truncation of an ideal single crystal. This is valid for the basal planes as well as for stepped or kinked surfaces. Therefore, the predictions should be compared to experiments that were performed on equally ideal electrode surfaces, e.g., single crystal electrodes. However, experimental surfaces always contain defects at the atomic level. Hence, theoretical calculation results can only be compared with samples that fulfill some quality criteria, usually inferred from comparison with stepped surfaces [2–9].

Additionally, in most of the catalysts, the ORR starts in a potential range, in which the surface is covered, at least partially, with some oxygenated species and, because the adsorption of oxygenated species may disturb the surface order [2–4], this limits the use of single crystal surfaces to understand the reaction mechanism on model surfaces. The latter aspect is especially important when comparing experimental data with theoretical calculations and creating idealized model mechanisms. Realistic model mechanisms are important because they would provide insights into the limiting steps, which could be further modified in order to enhance the overall activity of the reaction. Hence, it is essential that experiment and theory work together and assist each other to create a fundamental and strong basis about the knowledge of the reaction. It is worth to mention that, besides well-ordered monocrystals, non-contaminated working conditions, are necessary in order to get reliable data to compare and analyze against the ideal and perfect surfaces from simplified theoretical models [4,8–10]. Experimental work with non-ordered electrodes or slightly contaminated surfaces can lead to erroneous observations and conclusions.

In this presentation a critical view on the main recent experimental and theoretical findings about the ORR on Pt(111) and its vicinal stepped surfaces, in both acidic and alkaline media, is taken. The central idea is to find agreement and disagreement points that could serve to improve the current knowledge about the ORR surface reactivity. The motivation is the belief that only by building a common basis from theory and experiment we could progress in the understanding of this important reaction. The Pt(111) electrode was selected because this basal plane represents the most abundant facet on Pt nanoparticles, which are widely used as ORR catalyst in polymer electrolyte membrane fuel cells [11–13].

In addition, the role of the adsorbed oxygen-containing species and the possible relevance of the hydrogen peroxide oxidation and reduction reactions (HPORR) in the ORR mechanism are discussed. This is done specifically for high potentials, at which apparently there is no ORR current and the influence of the structure sensitivity of small particles appears [11–12,14]. This is relevant because only from a full understanding of the ORR kinetics it would be possible to unveil the identity of the rate determining step (RDS) on Pt, which would be an essential step toward an optimized design of new ORR electrocatalysts [15].

## Results and Discussion

**Figure 1:**
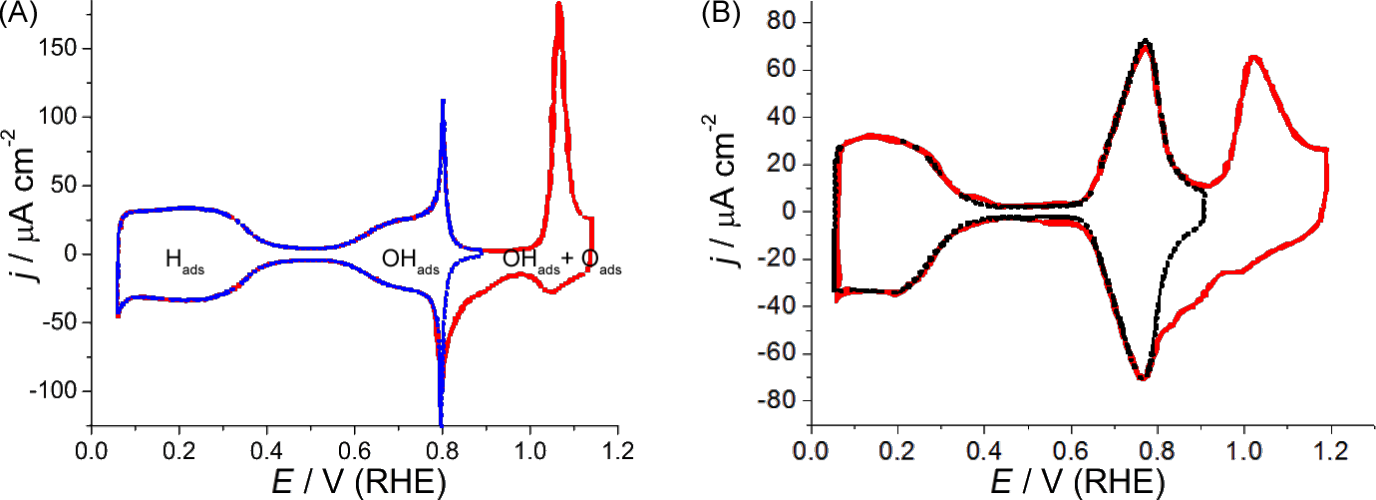
Stable voltammetric profile of a well-ordered Pt(111) electrode at 50 mV·s^−1^, at two upper potential limits: 0.9 V (blue or black) and 1.15 V (red) in 0.1 M: A. HClO_4_ (left). B. NaOH (right).

Ideally, the surface structure and composition of a catalyst remain unchanged over the whole potential range in which a probe reaction is scrutinized. However, as can be seen in Figure 1, this is not true in the case of Pt(111). At *E* < 0.35 V, hydrogen adsorption takes place, while at higher potentials, water can be considered the main species in contact with the surface. (In fact, water is always in contact with the surface regardless of the presence of adsorbates that arise from faradaic processes). In addition, if the electrode potential is increased beyond 0.6 V, the surface starts to be covered by oxygenated species. In this region, in acidic solutions the so called “butterfly” develops, which reflects the generation of PtOH, which is likely to happen in two steps [8–9]. In this case, the cyclic voltammetric profile (CV) shows a sort of passive region without significant current flow at higher potentials [6–8], until a well-defined peak starts to grow while an organized PtO adlayer is completed (1.0 < *E* < 1.15 V, Figure 1A [6–8]).

In the following we will mainly deal with acidic solutions, but we believe that the processes undergone in the butterfly and the following oxidation contributions are also likely to take place in alkaline solutions, although they have been studied less intensively (Figure 1B). Once the second oxidation is completed, the potential is close to 1.2 V and this is a strict upper potential limit (*E*_up_) that ensures the surface order of the Pt(111) electrode. In this respect, a good Pt(111) blank voltammogram would have no contributions at 0.12 V nor at 0.27 V, the hydrogen adsorption energy on {110} and {100} step sites, respectively [2–5]. Besides, it would not show any current contribution in the transition region from Pt–OH to PtO in HClO_4_ solutions [6–9] (Figure 1A) and negligible oxidation currents at potentials as high as 1.2 V in H_2_SO_4_ solution [2–3]. Even so, hydrogen adsorption has been proven to be less sensitive to the surface order than other probes, such as CO oxidation [10,16].

### Surface order

**Figure 2:**
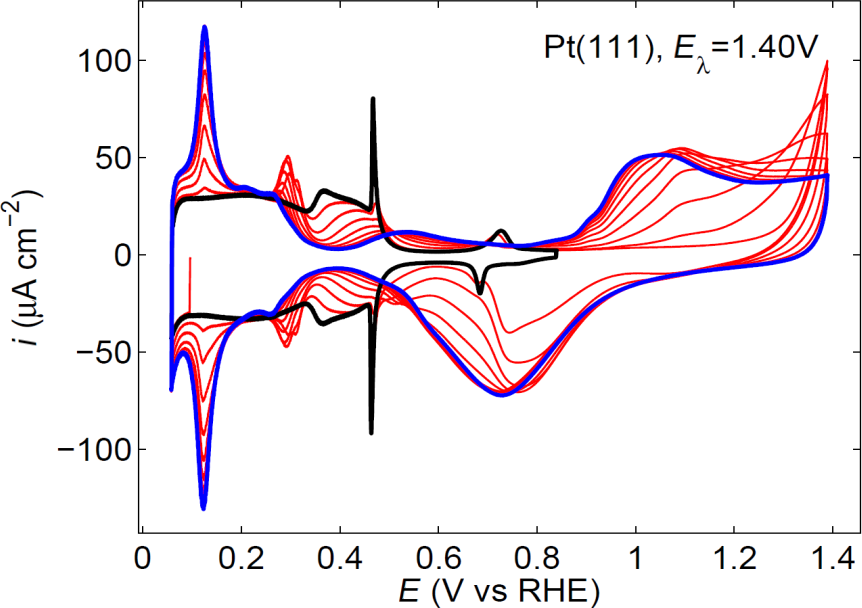
Evolution of the voltammetric profile of a Pt(111) electrode in 0.5 M H_2_SO_4_ as the electrode is cycled at 50 mV·s^−1^ between 0.06 and 1.4 V vs RHE. The black line shows the initial stable profile up to 0.85 V and the blue line the profile attained after 12 cycles (partially represented in red).

After potential excursions higher than 1.2 V, the CV in the immediate negative potential sweep shows the presence of {110} and {100} defects in the initially featureless hydrogen adsorption region, as a result of a surface reordering after the oxygen adsorption with high coverage (Figure 2) [2–3]. In this respect, the surface is uniform from the topographic point of view between 1.20 V and the beginning of hydrogen evolution, but the surface composition changes in the potential scale.

The surface disordering kinetics has been widely studied [2–4,7] and the charge density data have been well approximated by a consecutive reaction mechanism that is slightly influenced by an autocatalytic step [2–3]. With this procedure, it was observed that the disordering kinetics on Pt(111) is faster than on Pt(20,20,19), a surface with a 40-atoms wide {111} terrace [2–3]. This was an unexpected result that takes into account the existence of the autocatalytic step in the mechanism and points out the differences in reactivity between ordered defects and randomly generated defects. The process was more conveniently studied in sulfuric acid because the peaks associated to the charge of the “disordering products” at 0.12 V and 0.27 V, i.e., {110} and {100} defects respectively, are better defined in this media. Additionally, the decrease in the reactant domains can be easily followed by the decrease in the characteristic sulfate adsorption state at the {111} terraces [2–3].

With some limitations, the same procedure can be applied to non-adsorbing perchloric acid solutions, in which the adsorption peaks are broader, especially those corresponding to the {100} step defect [4]. In both cases, the surface disordering kinetics is faster as *E*_up_ increases, but the reordering rates are faster in perchloric acid than in sulfuric acid [2–4]. This result was expected and points out the effect of strongly adsorbed anions in the preservation of the metallic arrangement underneath. In contrast, when working with diluted solutions of sulfate anions (0.1 mM), it was remarked that the surface disordering is faster than in pure perchloric acid [4]. This is likely due to perturbations in the water network by sulfate anions, which in turn make the surface more vulnerable to the effect of the potential in the formation of surface oxides and the subsequent reordering of the surface [4].

**Figure 3:**
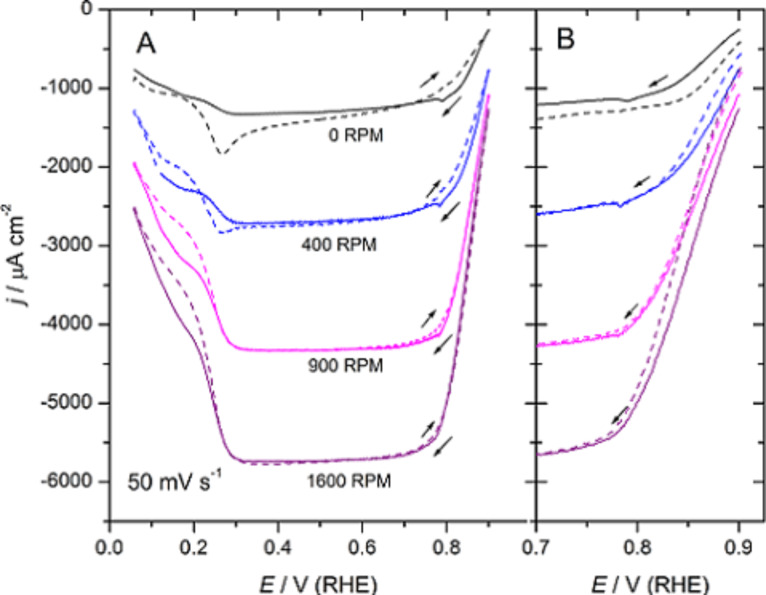
Oxygen reduction on a Pt(111) electrode in oxygen saturated 0.1 M HClO_4_. (A): Cyclic voltammetric profile: Positively (dashed line) and negatively directed (solid line) sweeps. The electrode was kept at the initial potential *E*_i_ = 0.06 V for 10 s before each measurement. (B): Negatively directed sweeps when the electrode was kept at *E*_i_ = 0.06 V (solid line) and at *E*_i_ = 0.90 V (dashed line) for 10 s before each measurement [18].

As it is pointed out above, models are idealized and thus state of the art experiments are required. In this respect the use of extremely clean solutions, under rotating disk electrode (RDE) experiments are necessary on well-ordered single crystal electrodes. Both Pt(111) spikes in perchloric acid (Figure 1A) and sulfuric acid (Figure 2) solutions should also be observed when the ORR is studied with the RDE configuration (Figure 3) [17–18]. Otherwise, the surfaces are probably contaminated with impurities coming from the solution that destroy these characteristic features. If both contamination and surface disorder appear, a fitting with theoretical models will be very difficult. In addition, if in this case both experimental measurements and theoretical calculations agree, one can consider that the tolerance range for the comparison is too large, and so a wide range of interpretations are possible. In order to set certain boundaries and to disentangle the details of the mechanism, it is important to use very strict experimental conditions.

### First stages of surface oxidation

In addition to the governing factors of the surface order, the surface disordering experiments showed some features in the CV of Pt(111) and its stepped surfaces that could affect the surface composition to a great extent, albeit the surface order is maintained. As a consequence, it was considered necessary to carry out a deep study on the first stages of oxide formation (PtO) at potentials higher than 0.95 V, but lower than 1.15 V. For this purpose, a carefully oriented Pt(111) electrode was prepared that had an “undetectable” level of defects in the hydrogen adsorption region. The starting point was a strict charge balance between the positively and negatively directed sweeps, which indicated that the solution was clean and free of oxygen [8]. These experiments [8] demonstrated the reversibility of the butterfly, even at high sweep rates. However, the next oxidation step, which is responsible for the peak at about 1.06 V showed irreversible characteristics (Figure 1A), and consequently the peak potential strongly depends on the sweep rate. It appears that under the envelope of this peak several processes take place that can be unveiled by modifying the potential perturbation program [8,19]. In the rising part of this peak, a nucleation and growth loop was identified in a limited potential range, together with a small reversible step. The latter step was similar to that observed earlier after flame annealing studies when the first potential scan runs in the positive direction from the rest potential [6]. This suggests that chemical reaction steps that involve the so-called thermal oxides could also give rise to a significant charge fraction of the peak at about 1.06 V [19]. The corresponding reduction process involves at least three steps, which spread over a wide potential range (Figure 1A). The most positive one can be considered more or less reversible, but it evolves rapidly to generate states that undergo a charge transfer at less positive potentials. This suggests that more stable surface species are formed with increasing time. The rationale of this second oxidation step after a stable surface state is attained, i.e. the butterfly, followed by a wide potential region in which no faradaic charge is transferred, was assumed to correspond to the formation of PtO from PtOH, as a phase transition that involves the adsorption of additional OH_ads_. This increased coverage of PtOH would destabilize the stable adlayer completed in the butterfly and generate other surface adlayers of varied composition, which finally generate a new relatively stable PtO adlayer at the end of the peak [8,19].

**Scheme 2:**
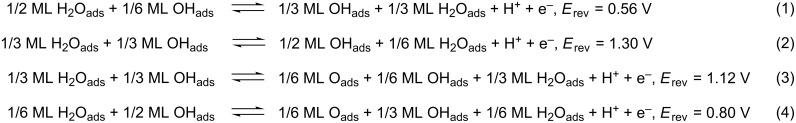
Possible adlayer reactions.

Theoretically, some possibilities have been suggested in order to illustrate how the different adlayers can be combined while the “total” oxygen surface coverage is increased as a charge transfer takes place [20–21] (Scheme 2).

The interconversion between these adlayers is evidenced by the different standard potential values of the different electrochemical equilibria as the oxygen coverage increases. In this scheme, the formation of PtOH at a higher coverage of that measured in the butterfly step seems to be the driving force [8,19]. Once PtO is formed, at the end of the positive branch of the peak at around 1.06 V, the stability of the adlayer increases. However, higher potentials would also generate higher PtO coverages that could further produce more oxidized forms. In these latter cases, however, the surface will start to disorder and would no longer be a flat, well-ordered Pt(111) surface.

### Oxygen reduction reaction (ORR) on Pt(111)

The cyclic voltammetric profile (CV) for the ORR on Pt(111), in 0.1 M HClO_4_, between 0.06 to 0.9 V, at room temperature and different rotation rates, ω, is well established (Figure 3A) [14,17–18,22,24]. In this case, the limiting current, *j*_lim_, is recorded between about 0.3 and 0.75 V and the reaction onset is ca. 1.0 V vs RHE [18]. It should be noted, however, that the limiting value is progressively reached between 0.7 and 0.3 V, in contrast to other diffusion-controlled processes, such as those of H_2_O_2_, see below. The appearance of two current drops at *E* < 0.3 V, at which the hydrogen adsorption begins, together with the detection of H_2_O_2_ [14] and a similar decrease in current in this potential region during the reduction of H_2_O_2_ [25], suggest that adsorbed hydrogen on the electrode, H_ads_, may prevent the O–O bond cleavage and block reactive surface sites. Therefore only two electrons are exchanged in this potential range [17,23]. This inhibition by H_ads_ depends on the surface orientation [14,17,23].

A kinetic analysis of the curves in Figure 3, either in the Tafel form or as Levich–Koutecký plots, suggested a first-order dependence with regard to the O_2_ concentration [22,24]. In addition Tafel slopes that range from 60 to 88 mV between 0.8 and 0.9 V have been reported [14,17–18,22–24]. In consequence, the first charge transfer step was proposed to be the RDS. Deviations of the apparent Tafel slope from its intrinsic value of 120 mV have been explained by using either a Temkin adsorption isotherm for ORR reaction intermediates [24] or by changes in the O_2_ adsorption because of changes in the coverage of chemisorbed oxygen-containing species [22–23].

**Scheme 3:**
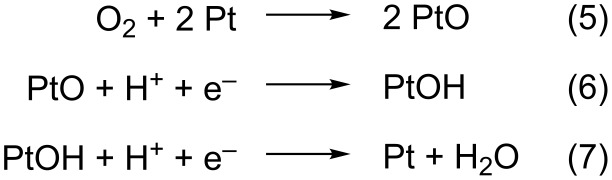
Associative ORR mechanism.

On the other hand, while the superoxide anion, O_2_^−^, has been detected in alkaline solutions as the ORR intermediate species [15], in acidic environments the picture is not that clear. In this media, H_2_O_2_ has been identified under some conditions as a stable ORR intermediate product [26–31], thus indicating an incomplete electron transfer. Nevertheless, the production of hydroxyl radicals, OH^•^, during the reaction has also been reported [32], and the reduction of the soluble hydroperoxyl radical, OOH^•^, as the RDS in the ORR in acidic solutions has been also suggested [18]. In contrast, other reaction intermediates have been suggested from quantum chemical models [33–42], and two main mechanisms have been proposed, namely the “dissociative” and the “associative” mechanism. In the first case, the O–O bond is broken upon oxygen adsorption on Pt. The simplest “dissociative” mechanism proposed for the reaction at a Pt(111) surface is shown in Scheme 3 [33].

**Scheme 4:**
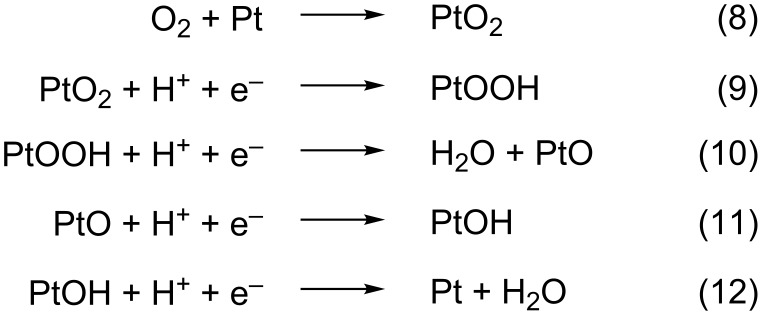
Dissociative ORR mechanism.

While in the associative case the O–O bond integrity is preserved upon adsorption and would only break after electron transfer (Scheme 4) [33].

The desorption of PtOOH to H_2_O_2_, which would react further at another surface site, instead of Equation 10 has also been proposed [34]. In this case, the associative mechanism can also be termed as “peroxo” mechanism [33]. In fact, this happens in experiments if strong adsorbates are present in the solution, such as strongly adsorbing anions or adatoms deposited at an underpotential [26,29–30,43]. In this case, H_2_O_2_ is detected as the final product in RDE experiments [14,26], which proves that the surface reactivity is important and that the availability of surface sites is a key point regarding the final reaction product.

It has been claimed that the reduction of OH_ads_ or O_ads_ is the RDS of the ORR [44–46]. Hence, because Equation 11 and Equation 12 are the same as Equation 6 and Equation 7, both dissociative and associative mechanisms can occur. However, the latter assumption would not directly lead to a first-order dependence on the O_2_ concentration nor to Tafel slopes between 60 and 88 mV. To account for these results, a theoretical model postulates that, together with site-blocking effects, OH_ads_ can alter the adsorption energy of ORR intermediates, and thus also have a negative energetic effect on the reaction [26,47]. However, OH_ads_ is also considered an intermediate reactive species in the H_2_O_2_ reduction (HPRR) on Pt [48–51], a mass-controlled reaction at potentials of up to approximately 0.95 V [25,49,51]. In consequence, the question about the real identity of the RDS in the ORR mechanism on Pt(111) still remains open. Especially because several studies have shown that improvements of the ORR at overpotentials were less than expected from the observed decrease of the OH_ads_ coverage [52–54].

### Surface sensitive reactions

Since early works [55–56], it has been known that there are volcano type responses when the ORR current densities, at a chosen potential, are plotted for different electrocatalysts as a function of either the adsorption bond strength, Δ*G*_ads_, of the O_ads_, OH_ads_ and OOH_ads_ species [55], or the electronic (Pt d-band vacancies) and geometric (Pt–Pt bond length) properties of Pt and Pt alloys [56]. Numerous theoretical calculations have supported this experimental fact and proposed a scaling relationship between the Δ*G*_ads_ values of these species that precludes any further improvements in the ORR performance, beyond some optimal values for these adsorption bond strengths, Δ*G*_OHads_, Δ*G*_Oads_ and Δ*G*_OOHads_ [33,44–45,57,59]. Similarly, for Pt(111) and its vicinal stepped surfaces a volcano type response for the ORR activity as a function of Δ*G*_OHads_ or Δ*G*_Oads_ has also been suggested, with the (111) facet at the top of this curve [13,60–62].

**Figure 4:**
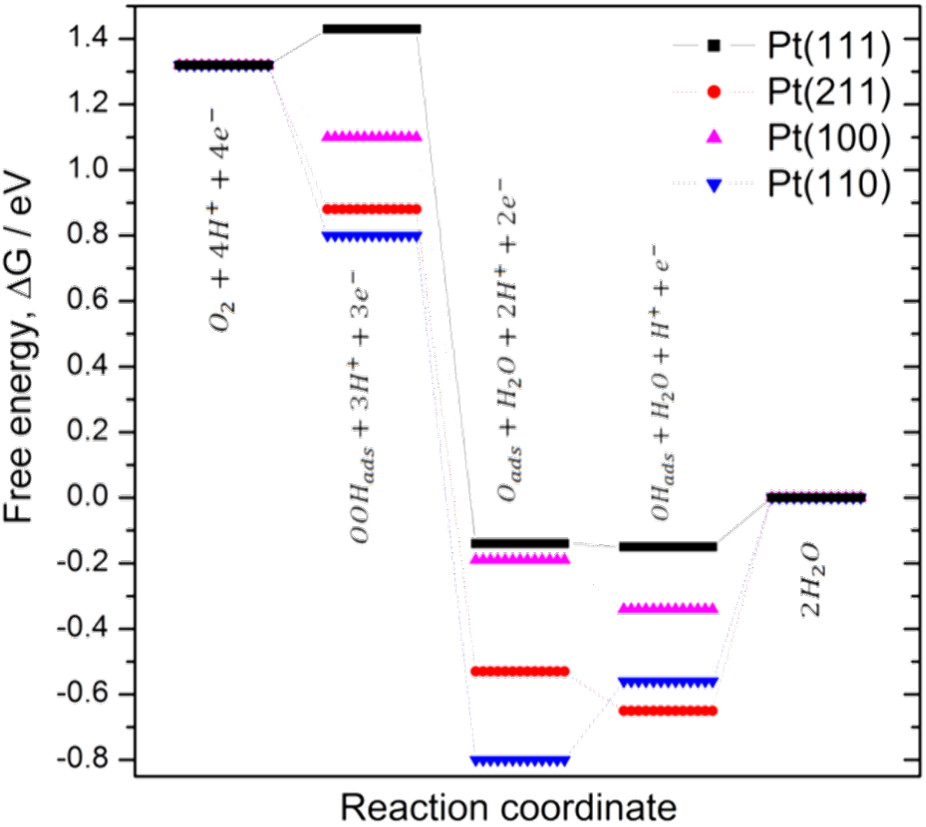
Potential free energy diagram for oxygen reduction over Pt(111), Pt(211), Pt(100) and Pt(110) at 0.9 V, on the basis of reported adsorption energies [13,33,44,58].

Following the same procedure reported in the literature and by employing the reported theoretical Δ*G*_OHads_, Δ*G*_Oads_ and Δ*G*_OOHads_ values [13,33,44,58], we construct the free energy diagram at 0.9 V (vs SHE) for the ORR at the Pt(111), Pt(211), Pt(100) and Pt(110) surfaces (Figure 4). In all cases, the used adsorption free energies were calculated while assuming a low oxygen coverage [13,33,44,58]. As can be seen from Figure 4, in the absence of any activation barrier the limiting elementary step for the ORR would be the OH_ads_ desorption, Equation 7 and Equation 12, because O_ads_ and OH_ads_ are relatively strongly bound to all Pt surfaces. Hence, it would determine the upper limit of the potential for the reaction to occur. The ORR activity sequence according this figure would be Pt(111) > Pt(100) > Pt(110) > Pt(211). Incidentally, if this were true, this would be bad news for practical applications because small nanoparticles cannot contain wide {111} domains.

In this picture, all the electron/transfer steps before Equation 7, or Equation 12, are downhill in terms of free energy. Exceptions are Equation 8 and Equation 9 on Pt(111) and Equation 6, or Equation 11, on Pt(110) but in these electrodes the OH_ads_ desorption has the largest positive free energy change in the whole mechanism. This simple picture is, however, not sufficient to explain the ORR mechanism, because the coverage of O-containing species at the surface changes with the potential and may affect the free energy of the different reaction intermediates [33]. In addition, adsorption energies in perfect UHV atmospheres may be different from those in aqueous environments, and hence, other elementary steps that are different from Equation 5 to Equation 12 may occur during experiments [18,32,38].

**Figure 5:**
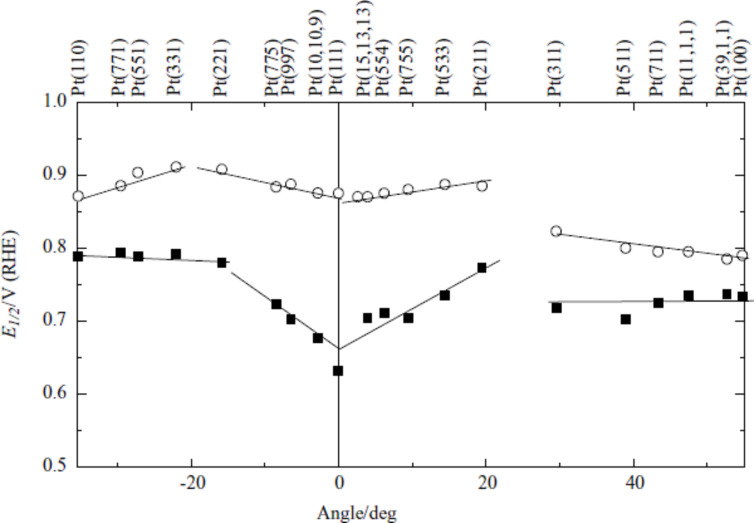
Plot of the half-height potential, *E*_1/2_, for the oxygen reduction as a function of the angle of the surface with respect to Pt single crystals, basal planes and stepped surfaces, in 0.5 M H_2_SO_4_ (filled squares) and 0.1 M HClO_4_ (open circles) [17,23].

In order to determine the surface sensitivity of the ORR, in our laboratory, we have approached the reactivity of the basal planes by extrapolation of the ORR activity from stepped surfaces with zero defects. In this respect, the assumption is that the steps “drain” defects in such a way that the terraces remain ordered. This was observed by STM, which shows that the defects seem to concentrate at the step lines and leave reasonably wide terraces between them [16,63–64]. Following this strategy, several experiments have shown that in acidic solutions Pt(111) is less active for the ORR than its vicinal stepped surfaces, irrespectively of the symmetry of the steps (Figure 5) [17,23]. This is in contradiction with the theoretical results described above [13,60–62]. Similarly, theoretical and experimental results for Pt(110) and Pt(100) also disagrees.

This result was carefully checked and confirmed [23,65]. Taking advantage of our facet-based surface orientation system, which allows small deviations of the angles from the basal plane, we prepared stepped surfaces vicinal to Pt(111) with long (up to 50 atom wide) terraces in the 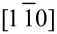 zone, which are considered to be the most stable surfaces for oxygen adsorption. The results showed again that Pt(111) is comparatively less catalytic for the ORR than the surfaces of the Pt(*S*)[*n*(111)×(111)] series [65]. This creates an inconsistent situation, because theory does apparently not fit with results that we believe are of the highest quality.

**Figure 6:**
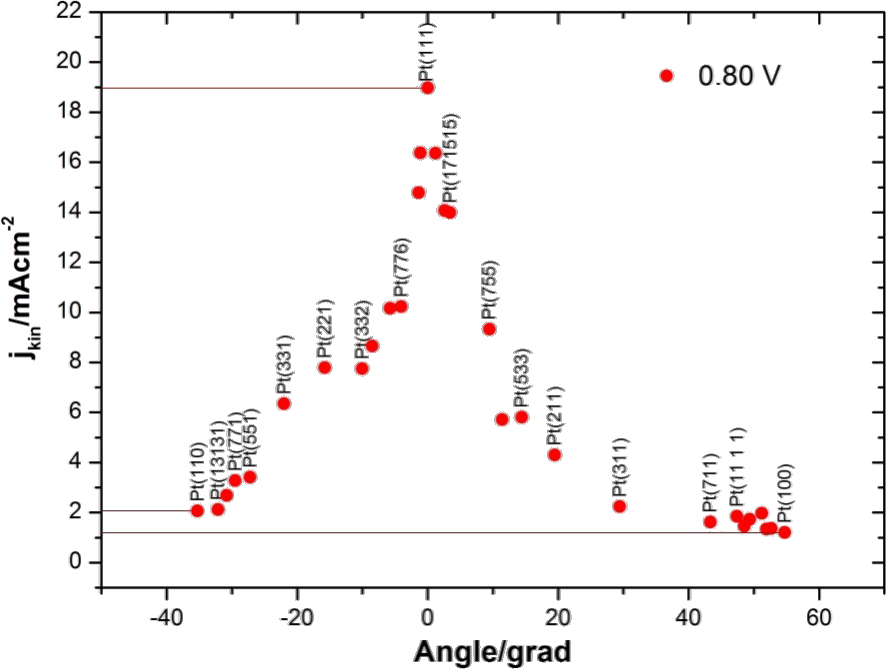
Plot of the kinetic currents at 0.8 V, *j_kin_*(0.8 V), for the oxygen reduction as a function of the angle of the surface with respect to Pt single crystals, basal planes and stepped surfaces, in 0.1 M NaOH [66].

This unsatisfactory situation ended when experiments were performed in alkaline solutions (0.1 M NaOH) (Figure 6) [66]. In this case, the overall results agree well with that expected from the theoretical “correct” trend, at least in the top reactivity surface, as the Pt(111) electrode becomes the most active plane. However, stepped surfaces were still more active than the other two basal planes, Pt(100) and Pt(110), in contradiction to what would be expected from theoretical calculations [13,33,44,58,60–62]. As mentioned before, this would be a problem for fuel cells, which would require large Pt nanoparticles. Fortunately, however, in alkali solutions Pt can be replaced by Ni and thus the main challenge in alkaline fuel cells is less the catalyst in comparison to the finding of a suitable membrane.

The interesting point is that the Pt reactivity predicted by the calculations involves a material with electronic surface charge densities that are about 0.7 eV more negative than those in HClO_4_, i.e., which is a difference of roughly 12 pH units. This raises the question of whether surface charges are appropriately included in the model. In relation to the available data, the potentials of zero total charge of Pt(111) are located at the beginning of the hydrogen adsorption and this potential shifts about 60 mV per pH unit [67]. This means that the metal side of the interface is positively charged in the potential range in which ORR starts, in both alkaline and acidic solutions that are free from dissolved species that strongly adsorb on the electrode surface and could interfere with species coming from water adsorption in the whole potential range. A cyclic voltammogram of Pt(111) in 0.1 M NaOH is depicted in Figure 1B.

However, a serious drawback in alkaline solutions deals with solution contaminants, which are more difficult to control than those in acidic solutions. This has been discussed in several cases and various interpretations were given. The first report on this problem was the result of a joint effort between Ulm and Alicante and was attributed to sulfate adsorption, while taking into account voltammetry and XPS experiments [68]. Recently, the problem was raised by Markovic et al. at the Argonne National Laboratory and it was considered to be because of metals like Ni, Co and Fe [69]. In our experiments, we have found these extra signals in the CV in some cases. To achieve the necessary voltammetric quality, it is necessary to use fresh chemicals (it is not possible to use the pellets or flakes some months after opening the flask) and solutions (daily prepared). Possible solutions to the contamination problem are currently under study in our laboratory.

### Limited ORR kinetics at high potentials

The surface changes during surface oxidation and disordering may be relevant in the ORR because of the electrocatalytic nature of this reaction, which involves adsorption steps. Results show that transport control appears at relatively high potentials of about 0.85 V (Figure 3A), but the ORR current differs between the positively and the negatively directed sweeps, the latter being higher especially at low ω (Figure 3B).That is the ORR current depends on the direction of the potential scan at 0.85 V < *E* < 0.9 V (Figure 3A) [18,23]. Initially, it has been suggested that surface oxides may be responsible for the discrepancies between positively and negatively directed sweeps around the onset of oxygen reduction [23]. However, under these conditions, the blank CV does not show any indication for the formation of PtO. Thus, only PtOH that comes from the reactions in the butterfly region could be expected to be on the surface, in equal amounts during both sweeps, because the butterfly is fully reversible at a sweep rate as low as 50 mV·s^−1^ [8–9].

**Figure 7:**
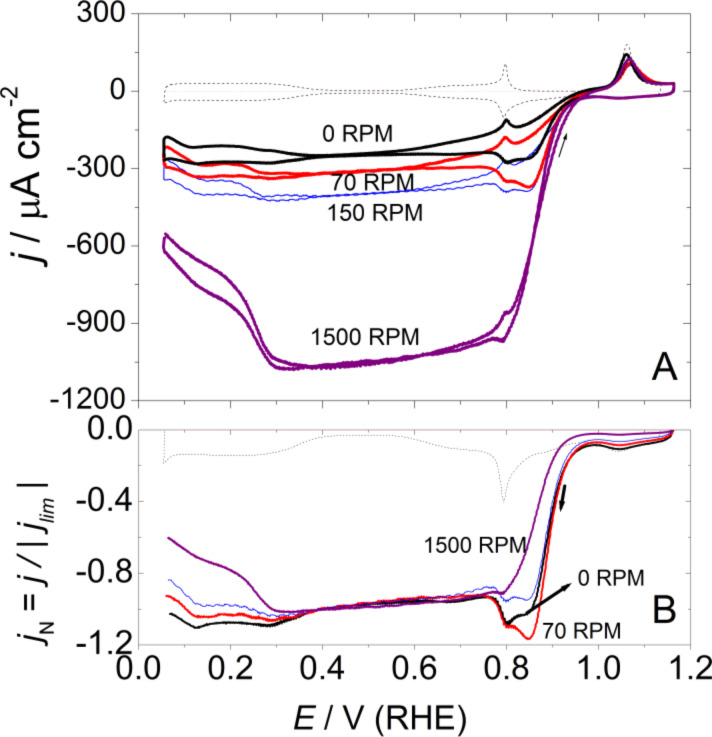
(A) Cyclic voltammograms for the ORR on a hanging meniscus rotating disc (HMRD) Pt(111) electrode from 0.06 to 1.15 V in 0.1 M HClO_4_, Ar/O_2_ ratio 5:1. Scan rate 50 mV·s^−1^. (B) Negatively directed sweep normalized against the limiting currents from data in (A).

**Figure 8:**
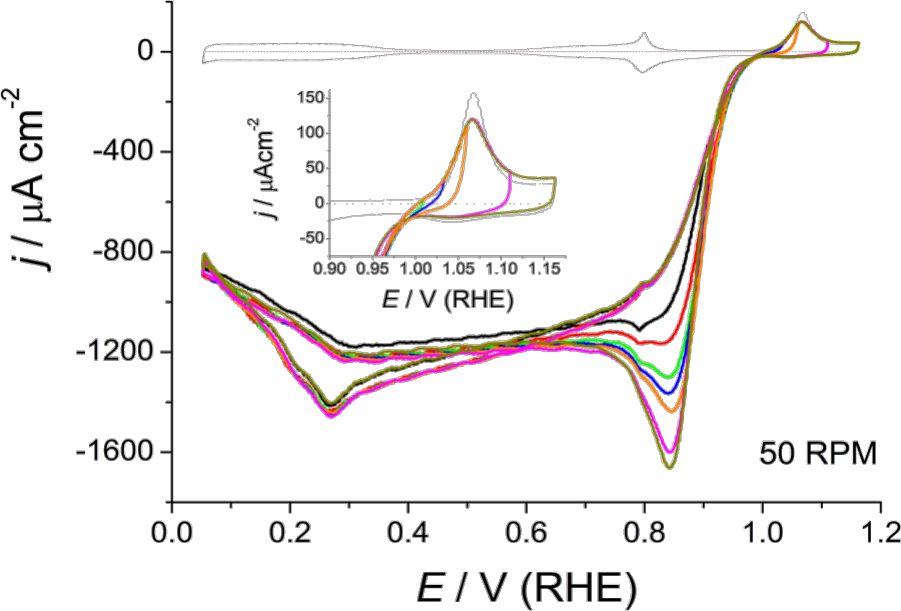
Cyclic voltammograms in the high potential region for the ORR on a HMRD Pt(111) electrode in oxygen saturated 0.1 M HClO_4_, at different upper potentials and 50 rpm. Inset: Detailed view of the Pt(111) oxide formation region.

To point out the possible effect of surface oxides on the ORR, experimental conditions can be chosen to minimize the influence of diffusional effects on the experimental response. This can be achieved by minimizing the oxygen concentration in solution and working at small rotation rates, ω. An interesting remark is that PtO formation and its reduction takes place at potentials at which the ORR just starts at Pt(111). In this respect, surface composition effects, if any, could be pointed out by comparing current densities in the positively and negatively directed sweeps in this potential region. Figure 7 shows the ORR curves at Pt(111) in 0.1 M HClO_4_, Ar/O_2_ ratio 5:1, at different ω, while Figure 8 depicts the ORR curve at 50 rpm for different upper potentials in an oxygen saturated solution.

Indeed, the presence of oxygen in the solution modifies the surface oxide dynamics, i.e., the current in the peak at about 1.06 V is decreased (Figure 7 and Figure 8) [18]. This experimental observation would suggest that molecular oxygen could participate in the formation of the adlayer, though at least one parallel path (or step) that could compete with more genuine electrochemical steps is already discussed [18]. This opens the possibility that the reverse would be also possible, i.e., the ORR could also be affected by surface oxides. Incidentally, the butterfly peaks, albeit clearly distinguished in the voltammogram, are slightly displaced (Figure 3 and Figure 7), but this could be an artifact of the combination of two independent processes. However, it should be remarked that the butterfly contribution should be observed, superimposed onto the ORR, at potentials close to its limiting diffusion-controlled value.

An interesting situation appears at high potentials with low ω and/or diluted oxygen concentrations. There is the presence of a peak, in the negatively directed sweep, with a reduction current higher than *j*_lim_ at *E* > 0.8 V, Figure 7A and Figure 8. When currents are normalized to *j*_lim_, it can be seen that this high potential current contribution progressively disappears as ω increases (Figure 7B). This would be compatible with the formation of a soluble intermediate species in the high potential region the concentration of which at the interface vanishes as ω increases [18]. The elimination of this soluble intermediate, however, is not very fast, which suggests some interaction with the surface. We can speculate that this interaction could likely take place through the water network, because of the necessary similar molecular composition of this intermediate and water, which would enable the formation of hydrogen bonds.

It is important to remark that the formation of this soluble intermediate is dependent on the potential, i.e., at low ω, the aforementioned peak contribution at high potentials increases at higher *E*_up_ (Figure 8). As the upper potential limit is not too high, this observation is compatible with the preservation of the Pt(111) surface structure. It should be remarked that the product, PtO, could be formed through a chemical process that involves dissolved O_2_, which could be faster than the equivalent electrochemical step that involves water. As a consequence, this complication should be considered in reaction models because it can be important in the formation of the soluble intermediate and lead to faster ORR at high potentials, which is the goal in electrocatalysis.

### Hydrogen peroxide oxidation and reduction reactions (HPORR) in the ORR

**Scheme 5:**
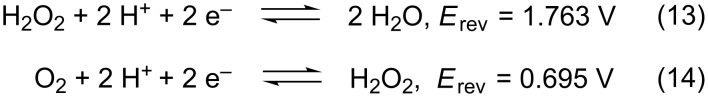
Reduction and oxidation of hydrogen peroxide.

From theoretical calculations, the single soluble intermediate species that could participate in the ORR mechanism is H_2_O_2_. However, as discussed above, H_2_O_2_ is a stable intermediate in ORR only under some circumstances [26–31]. It has not been detected in rotating ring-disc electrode (RRDE) experiments with either with massive Pt electrodes or Pt(111) electrodes in acidic solutions that contained moderately adsorbing anions provided that *E* > 0.35 V [1,14,22,26,70–71]. Hydrogen peroxide can be reduced and oxidized by following two different irreversible reactions that lead to water and oxygen, respectively, as final products according to Scheme 5 [25].

**Figure 9:**
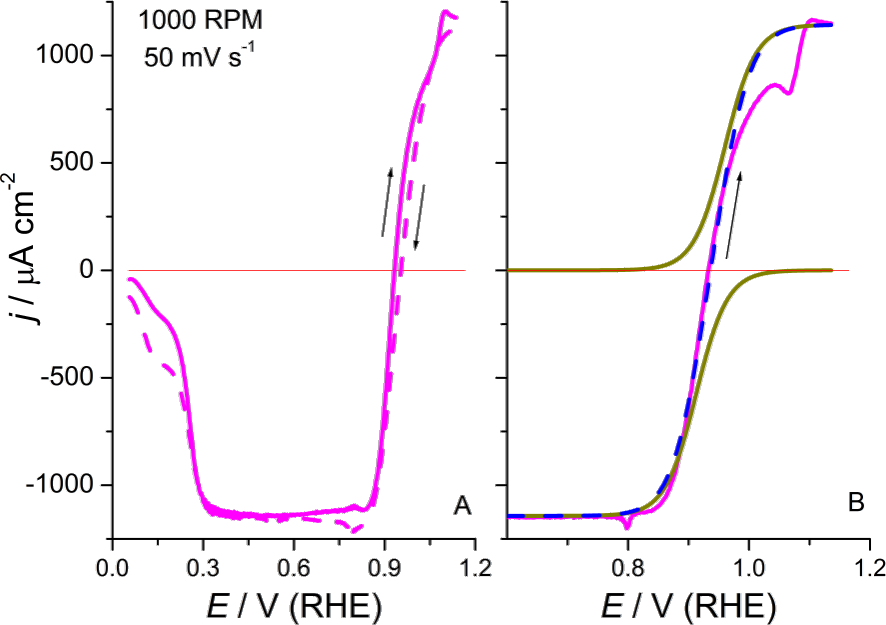
Hydrogen peroxide reduction and oxidation reactions on Pt(111) in 0.1 M HClO_4_ + 1 mM H_2_O_2_. (A) Cyclic voltammetric profile: Positively (solid line) and negatively directed (dashed line) sweeps. (B) Adjusted curves for HPOR and HPRR (dotted lines) during the positive scan. The dashed line corresponds to the sum of currents from the fitted branches and the solid line is the experimental curve after subtracting the blank.

It has been shown experimentally that the oxidation (HPOR) and reduction (HPRR) of H_2_O_2_ are fast reactions and that the total current is controlled by mass transport at any applied potential [49–51]. Hence, it has been proposed that the measured current is not resulting from the HPORRs themselves, which are considered to be purely chemical processes, but rather from the following electrochemical Pt-surface regeneration reactions [49–51]. Interestingly, on Pt(111) these reactions superimpose the CV in the potential range between the butterfly and the PtO-formation peak (Figure 9). In this potential region, the electrochemical activity is negligible in the supporting electrolyte solution, unless the upper limit becomes higher than 1.0 V at 50 mV·s^−1^.

From Figure 9, it can be seen that the HPOR and HPRR current densities reach the same *j*_lim_ with opposite signs. This corresponds to controlled diffusion processes that involve the same reagent and in two reactions involve the same number of electrons. The *j*_lim_ values agree with those expected from the Levich equation, within the experimental error range, which takes into account that in our experiments it is less important to use exact concentrations than to preserve the purity of the solution. However, in the upper diffusion limit the surface composition is PtO with an intermediate coverage whereas in the lower diffusion limit the surface is essentially water-covered Pt.

As in the ORR, if ω is not too high the butterfly peaks, as well as the PtO formation peak in the anodic branch, are clearly distinguished. The latter, however, is significantly reduced in charge (Figure 9A). Moreover, a clear distortion of the oxidation branch can be noticed as soon as the applied potential reaches 1.0 V (Figure 9B). This suggests that H_2_O_2_ can contribute to the formation of PtO through a chemical reaction, which is even faster than that by dissolved oxygen. It is interesting to remark that the inspection of the CV does not show important discontinuities when the zero current line is crossed, i.e., despite the different overpotentials for HPRR, about 800 mV, and HPOR, about 200 mV. The transition from oxidation to reduction and vice versa, which involves two different reactions, takes place without any apparent rate change in 0.1 M perchloric acid solution.

Because HPRR and HPOR are two different diffusion-controlled reactions, they can be analyzed independently under different experimental conditions in order to explore the relevant parameters that influence the mechanisms and to show similarities and differences. It is possible to arbitrarily decompose the HPORR by using conventional equations for S-shaped electrochemical processes given by


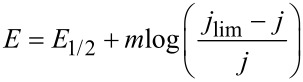


where *m* is a parameter that would depend on the particular charge transfer mechanism and *E*_1/2_ is the potential at which the current density, *j*, is one half of the corresponding *j*_lim_ value. Following this approach, a recent study in acidic media fitted the HPORR current potential curves on Pt(111) and vicinal surfaces, in such a way that the addition of both HPRR and HPOR contributions should agree as much as possible with the overall experimental curve [51]. It has been found that at oxide-free surfaces the structure dependence of the HPRR is similar to that of the ORR in acidic solution, while at oxidized surfaces the reactivity is comparable to what is reported for the ORR in basic media [51].

Figure 9B shows the adjusted curves, with *m* = 60 mV, for HPOR and HPRR (dotted lines) during the positively directed scan on Pt(111). The dashed line corresponds to the sum of currents from the fitted branches and the solid line is the experimental curve after subtracting the blank. It can be seen that the fitting is particularly good for the reduction process, which is almost unaffected by the surface oxidation, this assertion being also true for the negatively directed sweep (data not shown). In contrast, the HPOR is seriously inhibited in the potential range, in which *E*_2p,a_ appears in H_2_O_2_-free solutions. It seems that the formation of PtO affects the surface reactivity. However, once PtO is formed, the surface reactivity is restored and the reaction is transport controlled.

From the above, it is clear that at 1.0 V every H_2_O_2_ molecule that reaches the electrode will be readily oxidized. Hence, if the ORR takes place through the associative mechanism any oxygen molecule that could be reduced at potentials higher than 1.0 V, and thus yielding to H_2_O_2_, would be immediately re-oxidized to O_2_, while it is close to the surface. This would result in a zero net current until the HPRR becomes the dominant process, i.e., at *E* < 0.95 V. It is interesting to remark that in any case H_2_O_2_ would not be detected in the ORR under the present conditions, because it should be reduced as soon as it is formed with HPRR taking place at significantly higher potentials than ORR [25,49–51].

This opens the question about the identity of the soluble species suggested by the reported experimental results discussed above [18]. Recent theoretical calculations, which consider explicitly the effect of a bulk water layer on the mechanism of the ORR, have suggested that the dissociation of OOH_ads_, Equation 6, is much less favorable on the water covered surface as compared to the bare surface [38]. Hence, it would be possible that OOH_ads_ desorbs instead of being reduced and give rise to a soluble OOH^•^ radical species. Following these lines, next steps in the mechanism would be the aqueous reduction of OOH^•^


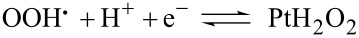


that will further reduce to water. Under this framework, the first–order dependence of the reaction, regarding the O_2_ concentration can be also explained. It is clear that this is only one possibility and more theoretical and experimental work is still necessary for a fully understanding of the ORR mechanism. However, the experimental shift of the oxygen reduction towards higher potentials described above should be kept in mind.

## Conclusion

In this work, a critical overview of the current state of the art of the oxygen reduction reaction (ORR) on Pt(111) and its vicinal surfaces has been done. Both experimental and theoretical results have been discussed and general points of agreement and disagreement highlighted.

It has been shown that despite the ORR shows a dependence on the surface structure, experimental and theoretical results disagree in acidic media while they seem to agree in alkaline solutions. The reasons behind this fact are not clearly known, but the necessity of building real and precise theoretical reaction models in order to get a fundamental knowledge about ORR mechanism is demonstrated. In contrast, the surface-structure dependence for HPOR and HPRR at oxide-free surfaces show similar trends as the ORR, despite that H_2_O_2_ is only produced during the ORR in acidic media when surface adsorbates are present.

The surface changes during oxidation and disordering of the surface may be relevant in the ORR because of the electrocatalytic nature of this reaction, which should involve adsorption steps. At high potentials dissolved oxygen may modify the oxide growth dynamics on Pt(111) and it is clear that both processes, ORR and oxide formation, interact. In this region, the reduction of a soluble intermediate species, different to H_2_O_2_, has been tentatively suggested from experiments as the rate determining step (RDS) for the ORR in Pt(111). However, there are no theoretical models that include both phenomena, yet and thus, the structures and processes at the molecular level that take place at the surface in this potential region, in which apparently there is no ORR current, are not clearly known.

More efforts are still necessary through a cooperative approach between theory and experiment in order to fully understand the ORR mechanism and to define the right approach for designing new electrocatalysts for fuel-cell cathodes. An agreement between theoretical calculations and experiments on model surfaces that describe the same process should be attained as a first step to understand the electrocatalysis at more complex surfaces such as those of dispersed nanoparticles.

## Experimental

In a similar way as described earlier [8], the working electrodes were prepared from small Pt beads, approximately 2–3 mm in diameter, by the method described by Clavilier et al. [72]. All the experiments were carried out at room temperature, approximately 22 °C, in a two-compartment, three-electrode all-glass cell, by following a well detailed experimental protocol [73]. Suprapure perchloric acid (Merck) and H_2_O_2_ (Panreac) were used to prepare the aqueous solutions in ultrapure water (Purelab Ultra, Elga-Vivendi, 18.2 MΩ·cm^−1^). H_2_, O_2_ and Ar (N50, Air Liquide) were also employed. All potentials were measured against the reversible hydrogen electrode (RHE) and a large, flame-cleaned, Pt wire coil was used as a counter electrode.
